# “If It Feels Right, Do It”: Intuitive Decision Making in a Sample of High-Level Sport Coaches

**DOI:** 10.3389/fpsyg.2016.00504

**Published:** 2016-04-14

**Authors:** Dave Collins, Loel Collins, Howie J. Carson

**Affiliations:** ^1^Institute for Coaching and Performance, University of Central LancashirePreston, UK; ^2^Grey Matters Performance Ltd.Stratford upon Avon, UK

**Keywords:** adventure sports, coaching practice, expertise, macro cognition, professional judgment and decision making, rugby

## Abstract

Comprehensive understanding and application of decision making is important for the professional practice and status of sports coaches. Accordingly, building on a strong work base exploring the use of professional judgment and decision making (PJDM) in sport, we report a preliminary investigation into uses of intuition by high-level coaches. Two contrasting groups of high-level coaches from adventure sports (*n* = 10) and rugby union (*n* = 8), were interviewed on their experiences of using intuitive and deliberative decision making styles, the source of these skills, and the interaction between the two. Participants reported similarly high levels of usage to other professions. Interaction between the two styles was apparent to varying degrees, while the role of experience was seen as an important precursor to greater intuitive practice and employment. Initially intuitive then deliberate decision making was a particular feature, offering participants an immediate check on the accuracy and validity of the decision. Integration of these data with the extant literature and implications for practice are discussed.

## Introduction

Intuition is of increasing interest to expertise researchers (e.g., Dane et al., 2012). Certainly, in the present context there is considerable anecdotal evidence that sports coaches often prefer to “go with the gut” (cf. Lyle and Cushion, 2010), taking fast action on the basis of what feels right (known as Naturalistic Decision Making; NDM) rather than through a more formal and slower Classical Decision Making (CDM; see Abraham and Collins, 2011) style reasoning which explicitly balances the options. Independently of how intuition is seen to operate (and various theories do offer different perspectives on the mechanism; cf. Klein, 2015), we were interested to build from our recent work in coach decision making (DM; e.g., Collins and Collins, 2012, 2013, 2015a,b, 2016b) to examine the role that intuitive decisions were perceived as playing in the repertoire of high-level coaches. Reflecting our interests and experience, and also to offer a contrasting pair of environments, we decided to conduct this primary exploration in adventure sports and rugby. Use of these settings enabled comparison of DM between a hyper-dynamic, perceived high-risk environment (adventure sports or AS; Collins and Collins, 2013) and a more conventional, less time pressured, and more ego than physical risk situation (at least for the coach!) such as the team sport of rugby union (RU).

Defined as “the capability to act or decide appropriately without deliberately and consciously balancing alternatives, and without following a certain rule or routine, and, possibly, without awareness” (Harteis and Billett, 2013, p. 146), intuition offers a significant extension to the concept of “on-action, in context” reflection described by Collins and Collins (2015a, p. 622) in their work with AS Coaches. Although, certainly with awareness and involving some balancing of alternatives, the on action-in context approach suggests an immediate review or “audit” of a quickly taken decision, offering the coach both a safety check and a reassurance that the gut feel action is correct.

The two environments used in this paper offer parallel but contrasting challenges for coach DM. The coaching of AS has emerged as a sub-set of coaching practice that draws on knowledge from the sports coaching domain and outdoor education (Collins and Collins, 2012). AS coaching practice is characterized by a high cognitive load brought about by the challenges associated with a hyper-dynamic coaching environment (Collins and Collins, 2016a). Specifically, the need to respond to challenges of a literally relentless, constantly changing physical environment, the infinite nature of the learners, and the relationship of the two provides a unique “wicked” challenge (Horn and Weber, 2007) to the coaching process. In our paper, examination of DM was considered across environments, but mostly focused on the majority environment, the teaching and development of clients in the AS situation, be it on the mountain, the river or the sea (cf. Collins and Collins, 2012).

In the more usual coaching environment of a team sport like RU, intuition is also an attractive construct. Decisions taken by high level coaches in the professional sport environment are certainly high stakes. Indeed, the high levels of challenge and turnover apparent (Cruickshank et al., 2014) make such environments equally high risk, albeit in a different way. In the data presented in the present paper, although once again all elements of DM were examined, comparisons were often drawn between teaching/training environments which, for RU coaches, also represent the majority of their coaching work with players. Environmental differences notwithstanding however (and these were carefully considered in our analysis), both these coaching settings impose high levels of challenge on coach–practitioners, offering an effective test-bed for our preliminary investigation of intuition in coach DM.

For practitioners in both, the implied expertise associated with intuitive or tacit DM has already been mentioned and even promoted in the coaching literature (cf. Lyle and Cushion, 2010). As such, there would seem to be some significant advantages to the adoption of intuitive DM for coaches, paralleling those already shown for performers (e.g., Janelle and Hillman, 2003; Raab and Laborde, 2011), so long as these were shown to generate decisions of equal (or even better) accuracy. Clearly, quicker and less effortful processing represents one big advantage: the possession of a knowledge base of sufficient richness to support/encourage intuitive DM is another concomitant benefit. Quite apart from the balance of advantage and disadvantage, however, there is a real need to achieve a more comprehensive understanding of coach DM.

In this regard, it is important to acknowledge the importance of research into professional judgment and decision making (PJDM) for the development of coaching practice and its status as a profession. Clearly, both the environments explored in this paper offer high stakes DM, with pressures varying from life and death (AS) through to professional rewards for success and sanction for failure (RU). Furthermore, understanding and effectively utilizing the cognitive and macro cognitive skills of coaches as the basis for assessment and professional development is increasingly recognized (cf. Collins and Collins, 2016b). In short, a move from a simple behavioral competency model to one firmly based in expertise is both overdue and would offer a significant step forward for coaching (see Collins et al., 2015). Certainly, the use of this approach would represent a definite move toward the recommendations associated with competence in professional settings (Kaslow et al., 2007). As such, examination of coach DM is of extreme utility to the profession, as well as an important topic in its own right.

In fact, research to date suggests that DM may take place on a continuum between CDM and NDM, with intuitive DM lying even further along the NDM end. The concept of nested DM (cf. Martindale and Collins, 2007, 2012; Abraham and Collins, 2011; Collins and Collins, 2015a,b, 2016b) as a part of the application of PJDM (op cit) to coaching, saw higher-order/longer-term decisions as best taken in a more considered deliberative (CDM) fashion while immediate, in-session decisions were more short-term and almost intuitive (more reflective of an NDM approach). The nesting of the latter within the former, so that short-term decisions generally took into account and catered for longer-term agendas, was suggestive that intuitive DM in a predominantly cognitive task such as coaching may show such an interaction.

Accordingly, and reflecting these different perspectives, we were interested to examine several pertinent aspects of coach DM as follows:

- the nature, scope, and incidence of intuitive DM in samples of high-level coaches;
- the relative frequency and origin of this DM style, at least as perceived by the sample, and;
- to what extent intuitive decisions interacted with more deliberative DM.

## Methods

### Participants

Participants were 18 male British coaches from rugby union (RU: *n* = 8; *M*_age_ = 48.2 ± 3.3 years) and adventure sport (AS: *n* = 10; *M*_age_ = 43.5 ± 12.5 years) domains. To ensure a sufficient level of domain expertise, experience, and inherent quality in terms of participants' self-reflective ability, purposive sampling was employed based on the following criteria: (1) a minimum of 10 years coaching experience since senior accreditation (RU: *M* = 14.9 years; AS: *M* = 15.1 years), (2) currently working with internationally-competitive and/or higher (e.g., professional/premiership) performers and/or hold the highest level coaching qualification within their respective sport, and (3) have a willingness to discuss their professional practice. All of the coaches were recruited through personal contact with the research team; the corresponding and second authors here being qualified and active practitioners within these two respective high-level sporting domains. This study was carried out in accordance with the recommendations of University of Central Lancashire's ethics committee with written informed consent from all participants in accordance with the Declaration of Helsinki.

### Procedure

To enable sufficient breath and richness of responses to be explored, a qualitative methodology was adopted. Specifically, semi-structured interviews were conducted with each coach in a quiet, private location, and at a time convenient to them. Participants received an information sheet by email at least 1 week prior to interview and, after consenting, the interview commenced by flexibly covering the lines of questioning shown in Table 1. In brief, the interview guide asked participants to recall and evaluate coaching episodes where DM utilized careful thinking (i.e., deliberative, CDM style) and others through sudden insight (i.e., intuitive, NDM style). In designing the questions, we were informed and guided by the work of Crandall and Getchell-Reiter (1993) whose application of the Critical Decision Method to nursing incidents in critical care offered a strong template. The classic and naturalistic types of DM were also explored more generally, as too were the learning experiences of each participant, and perceived skills, and attributes required to improve one's DM efficacy. Probes were deployed where necessary to gain additional information relating to interesting/important responses and/or check ideas against emerging literature, thus ensuring sufficient depth of response across all participants.

**Table 1:** **Interview Guide**.

**Question**	**Probes**	**Stimuli**	**Purpose**
1. Can you tell me about a recent coaching episode when you came up with a novel solution to a problem through careful thought?	• What did you do and with what aims?	• Performance improvement	• Explores incidence of reflective practice and “insightful experimentation”
		• Athlete/team development	
	• What did you believe were the key parts for achieving these aims?	• ID of the problem	
		• Generating and considering alternatives	
		• Experimentation?	
		• Criteria for PJDM	
2. Can you tell me about a recent coaching episode when you came up with a novel solution to a problem through sudden insight?	• What did you do and with what aims?	• Performance improvement	• Explores incidence of tacit knowledge
		• Athlete/team development	
	• Can you post hoc rationalize where the solution came from?	• Just popped into your head?	
		• Whilst coaching or away from the context?	
		• Something which was on your mind for a while?	
	• Can you think of any learning, knowledge or training which helped you generate this solution?	• Recent or past training, reading or viewing?	
		• Discussions with peers	
3. To what extent are your coaching decisions intuitive or carefully planned?	• Percentage incidence and context for each	• Try for a numeric balance across 100%	• Personal balance of styles
HOW and WHY?	• Personal preference	• Which do they prefer and why?	
	• Which do they think are most effective and why?	• Measure of efficacy they use?	
4. Where do you usually gain knowledge on coaching?	• Formal/informal balance	• Highlight previous successful learning	• Explores sources of learning
	• What is good about preferred approach	• Fits personal style	
Can you relate this to a recent change which you have made?		• The absolute best against theory	
		• In my experience	
	• What is bad about non-preferred approach?	• Inverse of above	
	• Have your preferences changed over time?	• Younger versus now preference and why?	
5. Can you tell me about any personal attributes and skills that you believe were important for carrying out the changes?	• Intelligence		• What does it take to be an innovator?
	• Open mindedness		
	• Critical reflection		
	• Adaptability		
	• Recognizing pivotal movements in the process		

Two researchers conducted the interviews and analysis of corresponding transcripts (see below), both are highly experienced in their respective fields and therefore were able to question, probe, and interpret responses with a degree of seniority. One of the researchers has 30 years of experience as an ASC at National Centers within the United Kingdom, is a coach educator, and holds Level 5 British Canoe Union coaching awards in four disciplines. The other researcher holds senior coaching qualifications in rugby, has experience of national level coaching in the United Kingdom and abroad, and has worked as a support professional in rugby at international level. Overall, the entire interview process lasted between 60 and 90 min. Data were recorded using a Dictaphone and stored electronically in mp3 file format.

### Data processing and analysis

Following the guidance provided by Braun and Clarke (2006), data were analyzed using a thematic analysis. Accordingly, interviews were first transcribed verbatim and each transcription was actively read several times prior to fully apprehend the essential features (Sandelowski, 1995). General impressions of these data were written in note form and shared between the two researchers conducting the analysis (first and second authors), highlighting any similarities and differences. Secondly, driven by an analytic interest of DM processes and informed by the literature, initial inductive coding of response data was applied to each transcript; thus formally identifying relevant and similar extracts. Thirdly, data codes were collated into potential lower-order themes based on common features, which were then grouped together under higher-order themes representing the highest level of abstraction. Within a fourth phase of analysis, these themes were subjected to review and further refinement. Meetings were held between the two researchers to discuss and compare the analysis between rugby and adventure sport domains. The primary aim was to check for a shared understanding and interpretation of data and, therefore, the emerging themes as a whole data set. This process was essential to detect genuine effective equivalence between situationally-specific behaviors; clearly the two groups were looking to generate rather different outcomes. As such, it was both informative and interesting that a high degree of overlap occurred. Our approach enabled themes to be combined and broken down, as well as the generation of new themes. Importantly, and reflecting our desire to examine genuine rather than artifactual (or even investigator created) overlap, the development of themes at any point during the analysis did not depend on the prevalence of a code, but rather, on what the theme revealed about the DM process. Finally, again as a collaborative process, the two researchers defined themes according to the essence of data codes within and how these might be perceived in relation to other existing theme definitions against the particular context of adventure sports or rugby.

In addition to the steps outlined above to ensure inter-coder agreement, the issue of trustworthiness was addressed through use of an additional researcher, who was not involved in the interviewing or coding process, independently coding a random sample of the transcripts (20%). This researcher coded raw data against the developed themes and his results were compared to those derived by the original process. Any disagreements (four emerged) regarding these differences in codes were discussed until a consensus was reached. This researcher also examined the overlap across domains, through interrogation of the equivalence derived from the thematic tables. Once again, disagreements (three emerged) were debated until a consensus was reached. Importantly, almost all of these disagreements fell within the first higher-order theme of learning environment.

## Results

A breakdown of the parallel thematic analyses are presented in Appendices A and B in Supplementary Material, with a summary table (Table 2) to exemplify points of equivalence and situationally specific difference. Unsurprisingly, differences were most apparent in the more situationally-specific settings of learning environment, although even here there was a very high degree of equivalence, most apparent in the intermediate, and higher-order themes arrived at by consensus across the research team. Reflecting the stated objectives of this paper, we report on a subset of the data yielded by the investigation, which predominantly draw on the second and third higher-order themes, which focus most specifically on our purpose. Following the structure outlined earlier, we report participant views relating to the three main objectives, followed by other relevant material from the interviews relating to the evolutions underpinning the use of intuition. For clarity and confidentiality, coaches are referred to by sport (RU or AS) and a number.

**Table 2:** **Summary comparison of key themes for adventure sport and rugby coaches**.

**Higher-order theme**	**Mid-order theme**	**Adventure sport coaches**	**Rugby union coaches**
Learning environment	Pedagogic context	10/10	8/8
	Environmental context	10/10	–
	Season context	–	8/8
Experience	Professional	10/10	8/8
	Reflective skills	10/10	8/8
Meta process	Aspects of decision making process	10/10	8/8
	Audit (change of perspective on DM)	8/10	8/8

### The nature, scope, and incidence of intuitive DM

All coaches recognized situations in which intuitive decisions were apparent in their practice. Coach AS2 articulated this as follows:

I think much more on my feet now. I'm much more intuitive as a coach than I ever was before. I come out with less and less structured [pre-planned] sessions. I've got structured sessions in the back of my mind, they're ingrained there, I've done them over and over again but what I do now is…I'll, kind of…I will adapt that. I will adapt that [the session] not only to the situation but also to the mood [the group's responses to his coaching] and to the environment as well.

This was echoed by coach AS9 who highlighted:

I prefer the one [DM style] that is right for the situation I'm in…I'd probably start with logical and linear, but there is a hell of a lot more intuitive that appears and it tends to go that way, because the likely course with a client, especially if it's long-term [characterizing the student's relationship with the coach], you're less likely to be following a logical linear path after a while. You're more likely to be reacting to what's going on around you, their development rate…So the linear journey to a goal might fall apart, especially in the long-term.

Rugby coaches were similarly universal in reporting that intuitive decisions fitted their behavior in certain settings. RU3 said “well I will take a gut feel and apply it, but almost always during a session or game.” RU6 added “we are surrounded with so much data on game day; but there is still a real part for intuition on substitutions, tactics, and the like.” Furthermore, all coaches acknowledged a role for intuition in player signing and selection. As Coach RU7 forcibly stated:

We get so much detail about what this guy is like…from agents, scouts, committee; f^***^, everybody gets in on the act. But at the end of the day, it's my call. And I make it almost entirely on feel…would I like to play with him, does he fit the [Club name] tradition, is he a good bloke as well as a player.

The intuitive characteristics of the DM process reported by participants appeared tacit (difficult to articulate) in nature but also to be based on refined and integrated reflective practice of a long, varied experience. As such, they emerged as aspects of a micro process, to meet a short-term challenge, but emanated from a longer-term macro process of development. Several coaches encapsulated meeting the challenges through intuitive decisions, but also highlighted the role of previous reflective practice as conferring an ability to *ad-hoc* rationalize a significant decision and unpack tacit aspects of the knowledge once utilized. AS5 described the decisions associated with observation of a group of whitewater kayakers prior to a session:

A lot of them [decisions] are sub…almost subconscious, that I don't quite know…, Like…, I don't sit there and go, right I've got five options that I could do with these people. It's more watching them on the water and thinking, well, what are they [doing].

AS4 encapsulated the gut feel as “I think it's how comfortable I am about sorting it out” when discussing a safety specific point. AS7 used similar descriptions of his decisions in relation to teaching and allowing the students to “…just let it happen, because I wanted to see how they [the students] would perform.” In both cases, the “gut feel” was to gain benefit (learning) from a risky situation (cf. an intuitive risk–benefit decision; cf. Collins and Collins, 2013). Both were confident that they could “sort out” the consequences so let things progress because “it felt right.”

Rugby coaches were identical in their reports of intuitive feel around running training sessions. RU2 spoke of how he would “let things run over, or change direction totally, going away from the plan to take a new direction just because it feels right.” Several spoke of how shared models and intuition with established colleagues enabled them to take new and novel directions in sessions with minimal or no discussion. RU4 reported on his long-term working relationship with another coach: “we just look across at each other, often without warning, nod and just both start working on something new; often in an entirely unplanned direction.” RU6 explained “I've been doing this for a long time…I think I coach now like a musician in a jam session!”

All these quotes are illustrative of a trait common to *all* the coaches interviewed; knowledge made usable and reliable in context by it becoming tacit following a period of reflection on extensive experience. The types of rule which emerged from this *post-hoc* reflection generates powerful tools for the future. Certainly, the use of rule-based strategies has been shown as a good way to handle unexpected or novel challenges (Richters et al., 2015). In this case it is interesting to see the ways in which rules may have emerged from initially intuitive action. Notably, however, this internalization or automation of earlier decisions, whether taken intuitively or through a more classical process, seems to occur through reflective processes; thinking through and weighing up the action before it is accepted as useful and locked into that individual's repertoire for subsequent, more intuitively led employment.

### The relative frequency and origins of intuitive DM

There were considerable variations, both between participants and between sports on the perceived frequency of intuitive DM. In all cases, participants acknowledged the need for careful planning across all elements of their work. Interestingly, however, the intuitive aspects of the coaches' DM emerged differentially across the macro and micro processes of the session. Within the macro process this could be observed in the planning stages of a session, and within the on-action/in context decisions that took place in the time generated for thinking within the session by the coach (cf. Collins and Collins, 2015a). Coach AS6 encapsulated the dilemma for the AS coach: “I suppose it would become intuitive if it's becoming something that you can't control from planning.”

For several, intuition seemed to be a feature of personal preference and professional context. Coach AS10 stated, “I think far more of it's rational than intuitive, but that's because I'm…. because of the nature of what I'm doing.” This contrasted with Coach AS5 who appears to state the opposite by explaining, “I'd say almost 90, probably 90% intuitive, 10% working out.” All AS coaches found difficulty in allocating a percentage to their DM process and qualified their original percentages when pushed. Coach AS5 qualified his original estimate by following his original quote (above) directly with “I'd say the working out was just at the beginning of the session.”

In contrast, rugby coaches were both more consistent and, perhaps, more conservative in their estimates of intuitive DM. As a typical comment, coach RU8 (the most senior and experienced of the sample) stated:

we tend to be quite rational and careful in planning…in thinking things through and justifying actions. Perhaps that is the team thing; we have to sell it to the coaching team and justify it to the players, especially when they are senior professionals. However, even the most staid coaches will, in my experience, take a leap in the dark sometimes; on a player, a substitution, a move or a change of plan.

As a consequence, perhaps, RU estimates of intuition DM percentage were on average lower, around 30%, with a much smaller range of 5–40%.

With regard to origin, participants were unanimous in acknowledging that their effective use of intuitive style had come with experience (cf. Pretz and Folse, 2011). Highlighted by Harteis and Billett (2013) as “the common elements of highly learnt procedures and informed strategic capacities that, together, support the capacity to act intuitively and with great effect” (p. 146), there seemed little doubt for our sample that they could now perform intuitively only because of a long and rigorous apprenticeship. Coach AS9 stated, “it's applying that decision making process in lots and lots of different situations over lots and lots of years in my case.” In similar fashion, RU4 explained:

when I started, I planned meticulously and almost agonized over decisions in case I got them wrong: but now I just go for it…I'm secure in my experience and can judge the situation as something I've seen before—so I can fly faster and with less thought—or something new which needs more careful thought.

This relationship, between situational awareness and the decision makers' experience and skill, contains the interactional aspect of the process already highlighted in our earlier work (Collins and Collins, 2016b).

The roles played by others, such as coaching companions, team members or, as stressed by the AS coaches, their community of practice (CoP), seemed particularly important in helping this group to the knowledge levels and associated confidence necessary for effective deployment of an intuitive style. Extending his ideas above, RU4 explained “I would have to credit my fellow coaches, my mentors, in building the knowledge and confidence which helped me “loosen up” and get intuitive.” All the rugby participants highlighted senior coaches (described as, but never formally in, a mentor role) as a major source of coaching knowledge and the support to make changes based on intuition; “to go with the gut” (RU3). Notably, however, self-directed reflective practice as a consistent strategy was far less common, with only two RU coaches describing this as an explicit and regular part of their development repertoire. CoPs, where reported, were perhaps understandably restricted, usually within the participants' club or to particular friends in the field.

In contrast, AS coaches all reported a high degree of personal reflective practice and engagement with their CoP: the role of the CoP being as a critical friend, a sounding board, and an exchange of coaching related knowledge. Coach AS10 highlighted “I think I'm lucky to be part of a community of paddlers” and further explained “I get to see and hear other peoples' [coaches] perspectives.” Coach AS4 described the characteristic of a productive CoP working in mountainous artic conditions; “it's very much a supportive culture, people are quite happy to ask about advice and some is a bit wacky [the advice] and some will say, I don't think you should go there today.” Coach AS5 described the characteristics of an effective CoP with a coaching focus; “Yes. Yes, definitely. Yes, just willingness for everyone to go, “oh, you do it that way and you do it that way,” or not…not having any fixed…fixed way of doing it.” Notably, the seven AS participants involved in coach and leadership education, in addition to their skills development role, were able to articulate and clearly value their DM knowledge and skills originating from regular CoP interactions.

### The interaction of intuitive and other DM styles

As intimated in several of the quotes above, participants were very aware of the parallel and/or interactive use of intuition with other, more deliberative styles of thinking in their coaching. Interestingly, they reported very few examples of intuition as a quick “that will do” but suboptimum alternative (cf. fast and frugal; Gigerenzer and Todd, 1998). All the coaches in this study recognized both intuitive and classic characteristics in their DM processes during coaching. A sizable minority treated the two as somewhat distinct, suitable for use in certain circumstances. As RU2 reported:

I think it reflects my original profession [uniformed services]. I recognize a situation as requiring decisive decisions and get myself in the headset to act so. This almost always includes making big calls on feel. I do debrief them later but, in the moment, its card laid, card played!

In similar fashion, coach AS6 stated “I think if done right they're both effective,” referring to considered and intuitive processes although implying a non-nested relationship. Coach AS7 linked the characteristic of the DM process to other aspects of practice and clearly illustrated a comprehension of the characteristics of his DM process:

I would like to say, when I was learning and gathering my experience, it's definitely that planned [considered] approach because you felt safe, you felt okay…Whereas now [referring to intuitive characteristics], I kind of, almost get a bit more excited, I didn't expect that, that's great. Let's go with it and see how it goes, probably because you know at the back of your head, that if it starts to go wrong, you can still fix it and put it back on track.

This appears to illustrate a necessary confidence in the intuitive elements that has emerged over time and practice. These coaches declared personal preferences for a given approach, with the preference reflecting their experience and personality; importantly, the coaches all articulated a confidence in being more intuitive or considered because of an imbedded audit of the decision making process.

Going further, however, a majority (8/10 AS and 4/8 RU) demonstrated an additional meta-process in the more integrated use of the different styles, based around a clear recognition of the particular advantages or disadvantages in that context. This idea resonates with the “rich systematic interactions” identified by Christensen et al. (2016, p. 40) as crucial between automaticity and cognition in movement execution. Under this “Mesh” approach, athletes exhibit a delicate but consistent balance between cognitive and automatic elements of control, except when the balance is disrupted (often by anxiety) toward an overly cognitive style. The relationship between the intuitive and classic aspects of DM in our participants was nested in nature and influenced by two factors; a context-based, situational awareness and the decision maker's experience and skill. For example, AS5 suggested he chooses from “millions of options” and recognizes “I do like a coaching problem.” In all these cases, the DM process, including a refined reflective practice, was imbedded within the coaching process and audited by the coach.

These 12 coaches articulated an *ad-hoc* triangulation/audit of the DM process that was achieved via a notional question: “is the same outcome achieved via a different DM approach?” The audit was used to verify or challenge the original decision which informed action on a particular course of action. This triangulation/audit is time consuming and adds to the time pressures but was considered valuable given the complexity of the environment and consequences in sport. This audit was integrated into the process by creating time for DM, not just for the original decision but also for the audit (Collins and Collins, 2015a). As RU1 observed “I decide to do something, say make a substitution, but immediately I'm scanning the decision to see if it feels right.” These descriptions fit well with the parallel systems ideas of Myers (2002) and Sadler-Smith (2010), representing the twin use of intuition and deliberation to generate optimum solutions.

Interestingly, however, and perhaps representing an extension to the parallel systems ideas, this audit process did not necessarily use the alternative deliberative style but was often also intuitive in its nature. Often, the use of intuition to audit intuition was determined by pressure; from the environment, context or, most notably, emotions (cf. Slovic et al., 2002). It was in these situations that intuitive skills appeared most valued by the coaches. AS4 described a forced decent from a winter climbing route in deteriorating conditions in which his decision “was the least of all bad options, there weren't many…there was no good option and it was the least of all the bad options, there were no good options really.” He later described the decisions as needing to “go with your gut” (the primary, let's retreat decision) while asking a rhetorical question of himself “does this feel right” as the auditing process for the route selected. In similar fashion, RU5 reported “in that situation I felt really angry. I wanted to take action so made the call, at the same time thinking to myself “does this feel right”?”

The work of Eraut (1994, 2000) offers a very parsimonious explanation of our data on style integration. As he states, intuitive responses may be represented as:

not only pattern recognition but also rapid responses to developing situations…based on the tacit application of tacit rules. These rules may not be explicit or capable of reasoned justification, but their distinctive feature is that of being tacit at the moment of use (Eraut, 2000, p. 127).

AS4 articulated this challenge in needing to rely on easily accessible decision making skills and demonstrates a need for confidence in the NDM process together with a realization of a meta-process that exists within the NDM aspects of the process. AS4 was torn between gut feel and recognition primed DM, and a capacity to articulate the complexity to his students:

….trying to convince the students [articulating the dilemma] that that was a really serious day and the decision making, they all thought it was fantastic and it was a really exciting adventure, but you know, it's trying then to tell the students, actually…there was some wrong decision making going on there. There was some gut decision making that was…that basically was fine up to a certain stage [limitations of a given approach], but then it's the conditions and the environment changed [situational awareness, change, and impact] and so I was stuck, having to make gut decisions [other processes may have been better suited], and realizing that I was now in a situation that wasn't good [audit].

*Post-hoc* recall and rationalization of decisions, however unconscious/intuitive, was a common feature across participants. As RU1 observed:

In the heat of battle, I say and do all sorts of things. My coaching team and analysts often look at me strangely to think “why the f^***^ has he done that?” But I can always run the replay in my head afterwards, with total recall, and explain the logic of why, when, and how even though I wasn't aware of it at the time.

In summary, participants showed individually consistent but, inter-individual, variation in the types and degrees of integration between intuitive and deliberative styles. Whether this is truly indicative of parallel processing is for the moment, beyond the reach of our data.

## Discussion

While the existence and use of intuition as a DM approach was clear from our data, when and how it was used is of greater interest, especially when contrasted to the application of more deliberative strategies. Clearly, the proportion of considered to intuitive characteristics in a given decision varied depending on context, based largely on the coach's high situational awareness of a given session or context (Endsley, 1995a,b). This awareness seemed to be situated within practically set parameters that framed the process, and included elements that are managed by the coach (e.g., logistics, equipment, student/player preferences, fitness, cognitive ability) plus, potentially, learning outcomes, (i.e., an outline plan), and some that cannot be directly managed (e.g., tides, snow conditions, weather or player reactions, game outcomes, etc.). The role of the *post-hoc* audit check (whether quick or more deliberate) is another crucial finding for future work; intuitive decisions in coaching may not be as unaware as Harteis and Billett (2013) suggest. Such an action is understandable in the hyper-dynamic, high stakes environment of AS. Finding it in the more conventional and less (comparatively) time pressured world of mainstream RU coaching (the majority of time, and our data, came from the training environment) however is, perhaps, more surprising. This idea merits more detailed examination.

Extending this interactive theme, it is worth considering more carefully how the relationship between deliberate and intuitive DM may operate. This relationship is clearly “perceptual” in its nature and operates continually within the coaching process, forming the coaches' awareness to the situation (situational awareness) as it evolves. In the present sample, this appeared to be related to three interacting contexts. The first, the pedagogic context, appears to be comprised of a further set of sub-factors such as the learning outcomes, syllabus/issue content, potential goals, and the nature of the individuals being taught. The second environmental context relates to the real and perceived risk to the participants by considering the physical and social environments of the decisions. In rugby, though less serious, this session context is also clearly important. These two, the pedagogic and environmental/situational content, interact to form a third subgroup, the learning environment, which links to the decision maker's experience and skill in DM. Our suggestion here is that effective use of an intuitive DM style, indeed all styles, will be determined by the education which the coach receives on how coaching works (cf. theories of knowledge generation; Nonaka and Takeuchi, 1995). Accordingly, and particularly from an evolutionary perspective, coach educators may also need to consider the environment in which such evolutions may be optimized (e.g., Nonaka and Nishiguchi, 2001).

Irrespective of how they may best be developed, intuition and analysis are both important components of expertise and their mutuality seems well supported by our data (cf. Pretz, 2011). Interestingly, this interplay seems to be most important in certain settings and conditions. In AS coaches, for example, the interplay of considered and intuitive characteristics form part of the coaches' ability to rapidly adapt and be flexible in session, this appears to be facilitated by the intuitive aspects of the process. The in-action decision process that requires greater flexibility and adaptability having more intuitive characteristics. Adaptability and flexibility (modification of existing knowledge) was considered by all the AS coaches as an aspect of their expertise. A lower number of AS coaches (*n* = 7) identified creativity (creation of novel solutions in-action or on-action/in context) as an aspect of their expertise. In this respect, our own rule for the use of new information in a coaching situation involves an immediate scan and placement as follows…“Act on, Store or Ignore”: in short, making a rapid initial evaluation on the potential worth of new data. This enables the essential rapid action within the hyper-dynamic environment of adventure sports; without it, the coach may quite literally drown in data and be paralyzed by in-action reflection.

The position of rugby coaches was somewhat different on the first aspect, most particularly because their DM was usually less time pressured and dynamic than AS. As such, the interaction with deliberative DM was more marked and frequent. There was much greater concordance on creativity, however. Almost all (*n* = 7) of the RU coaches mentioned the role of intuition in generating novel and creative solutions to problems they encountered. Clearly, the aspect is particularly worthy of examination, especially at the top end of performance where originality is often key to success.

Another important issue for the future relates to how intuition might best be investigated. We acknowledge the comparative crudeness of our “percentage of intuition” question (Q3 Table 1); also the limitations inherent due to small numbers in our sample, albeit these participants are of a high level and hence, drawn from a small population. Our point is the self-reported difficulty participants experienced in answering the question, which means that future studies must use tracking to generate more accurate figures. We see the present study as a first exploration and, as such, report the data accordingly. For the future, however, in our own and other's work, how this tracking is best accomplished is an issue. It is not, we suggest, just a case of “think out loud” (cf. Whitehead et al., 2015) although our data suggest that an immediate internal or even external audit often follows a gut feel decision. Perhaps the best option is a more naturalistic observation of the process, with immediate follow up and critical probing to take the participant back through the situation soon after it has occurred. The instance-driven interview questions used in this study (see Table 1) are an NDM research technique that can/should be used to operationalize Intuitive DM in ways that can be easily interpreted and subsequently included in coach education initiatives, so long as the *post-hoc* elements described are also tested for. We have certainly used this approach to good effect in our own work on coach DM (e.g., Collins and Collins, 2015a,b, 2016b). In any case, methodological issues will be an important consideration for the future.

We also need to investigate the evolution of intuition in coaching, especially since its popularity and high credibility status (cf. Tetlock, 2006) may “encourage” such habits in beginner coaches. After all, peoples' preference for decisiveness has been well documented! Our participants were certainly supportive of the conventional wisdom that intuitive DM emerges from experience. Once again, however, research from nursing offers some interesting parallels and contrasts. For example, Ruth-Sahd and Tisdell (2007) suggest that the use of intuition is more related to previous experience with it as a style than level of training. Others see the use of intuition as a trait (e.g., Myers et al., 1998; Pacini and Epstein, 1999). These issues notwithstanding, there seems to be a strong case for intuition as a characteristic which emerges from experience (cf. Pretz and Folse, 2011) and, as such, more advanced training may well-include its use as a consideration using suggestions from the NDM literature as a basis (cf. Klein, 2015).

There is one other issue worthy of note; that is, against the definition used of intuition as an unconscious act, our data seem to suggest that the process (at least as seen by our coaches) is often semi-conscious or, even if unconscious at the time, almost immediately brought into the conscious space and rapidly reviewed. Certainly, several of the studies cited in this paper have highlighted this conundrum (most notably the nursing research). Is there, perhaps, the need for a new model with regard to the definition of intuitive thinking? We see interesting and important parallels between these ideas and the Mesh control suggested by Christensen et al. (2016) as a parsimonious solution to the interplay of conscious and automatic processes in movement. This issue awaits further examination. For the moment, however, the place of intuition in the DM of high-level coaches is clearly established, albeit that it might be less automatic and implicit than some popularist authors may suggest.

## Author contributions

DC developed the study concept and design of the work. DC and LC were responsible for data acquisition and all authors were involved in the analysis and interpretation. DC and HC prepared a draft of the manuscript; all authors provided critical revisions to the final submitted version and gave approval for it to be published. Finally, all authors agreed to be accountable for all aspects of the work in ensuring that questions related to the accuracy or integrity of any part of the work are appropriately investigated and resolved.

### Conflict of interest statement

The authors declare that the research was conducted in the absence of any commercial or financial relationships that could be construed as a potential conflict of interest.
