# Supplementary material for: “If It Feels Right, Do It”: Intuitive Decision Making in a Sample of High-Level Sport Coaches
Source: Front Psychol. 2016 Apr 14;7:504. doi: 10.3389/fpsyg.2016.00504 (PMC4830814; doi:10.3389/fpsyg.2016.00504)
Supplement: Supplementary file 1 [file DataSheet1.docx]

Appendix A. Thematic table of responses for AS coaches as derived from the inductive analysis.

| **Higher-order Theme** | **Mid-order Themes (Interrelated)** | **Lower-order Themes** | **Coaches** | | | | | | | | | |
| --- | --- | --- | --- | --- | --- | --- | --- | --- | --- | --- | --- | --- |
|  |  |  | **1** | **2** | **3** | **4** | **5** | **6** | **7** | **8** | **9** | **10** |
| **Learning Environment** | **Pedagogic Context** | Logistics (transport, equipment) | × | × | × | × | × | × | × | × | × | × |
|  |  | Knowledge of student needs and wants (observation, questioning, benefits) | × | × | × | × | × | × | × | × | × | × |
|  |  | Knowledge of student ability (observation, questioning, benefits) | × | × | × | × | × | × | × | × | × | × |
|  |  | Learning outcomes (needs and wants, syllabus constraints) | × | × |  | × | × |  | × | × | × | × |
|  | **Environmental Context** | Weather (past, present, and future) | × | × | × | × | × | × | × | × | × | × |
|  |  | Conditions (tides, snow, wind, inter-relationship) | × | × | × | × | × | × | × | × | × | × |
|  |  | Real risk perceived by coach and student | × | × |  | × | × | × | × | × | × | × |
| **Experience** | **Professional** | Decision making in own adventure practice | × | × | × | × | × | × | × | × | × | × |
|  |  | Role (Guiding, coach, coach education, education) |  | × | × |  | × | × |  | × |  | × |
|  |  | Time to think (pedagogic, practical strategies, opportunistic) | × | × | × |  | × |  | × | × | × | × |
|  |  | Explicit/Tacit | × | × |  | × | × | × |  | × | × | × |
|  |  | Decision making (see meta process) | × | × | × |  | × | × |  | × | × | × |
|  |  | Reflective process (see Reflective skills) | × | × | × | × | × | × | × | × | × | × |
|  | **Reflective Skills** | Community of practice |  | × |  | × |  | × | × | × |  | × |
|  |  | Coaches skill as reflective practitioner | × | × | × | × | × | × | × | × | × | × |
|  |  | In action (intuitive basis to reflective practice) | × | × |  | × | × | × | × | × | × | × |
|  |  | On-action (Classic basis for reflective practice) | × | × | × | × | × |  | × | × | × | × |
|  |  | On-action/in context (create time to think, pedagogic, practical, and opportunistic strategies) | × | × | × | × | × | × | × | × | × | × |
|  |  | Pre-action (aspect of planning, creation of contextual framework for decisions in action) | × | × | × |  | × | × | × | × | × | × |
| **Meta Process** | **Aspects of Decision Making Process** | Considered process/planning (pre-action, on-action, creating time) | × | × |  |  | × | × |  |  | × |  |
|  |  | Benefits of proposed action (i.e., a risk vs. benefit decision) | × | × | × | × | × | × | × | × | × | × |
|  |  | Intuitive process (on-action/in context, creating time, opportunistic) |  | × |  | × |  |  |  | × | × | × |
|  |  | In session (in action and on-action/in context, nested) | × | × |  | × |  | × |  | × | × | × |
|  |  | Adaptability/flexibility | × | × |  | × | × | × |  | × | × | × |
|  |  | Creativity | × | × |  | × |  | × |  | × |  | × |
|  | **Audit**  **(change of perspective on DM)** | Meta decision. “How best to make the decision?” |  | × |  | × | × | × |  | × |  | × |
|  |  | Personal preference, pros and cons (macro and micro process) | × | × |  | × | × | × |  | × | × | × |

Appendix B: Thematic table of responses for RU coaches as derived from the inductive analysis.

| **Higher-order Themes** | **Mid-order Themes (Interrelated)** | **Lower-order Themes** | **Coaches** | | | | | | | |
| --- | --- | --- | --- | --- | --- | --- | --- | --- | --- | --- |
|  |  |  | **1** | **2** | **3** | **4** | **5** | **6** | **7** | **8** |
| **Learning Environment** | **Pedagogic Context** | Logistics (equipment, surface condition/quality) | × | × | × | × | × | × | × | × |
|  |  | Knowledge of player needs and wants (longer-term program aims, short-term game agendas, new skill sets needed) | × | × | × | × | × | × | × | × |
|  |  | Player skill level/playing level (i.e., capacity for learning) | × | × | × | × | × | × | × | × |
|  |  | Team needs – factors influencing on outcomes (next game vs. schedule vs. season, need for players) | × | × | × | × | × | × | × | × |
|  | **Season Context** | Immediate challenge of upcoming opposition | × | × | × | × | × | × | × | × |
|  |  | Upcoming games – influence on selection, etc. | × | × | × | × | × | × | × | × |
|  |  | External challenges – Six Nations, Internationals, etc. | × | × | × | × | × | × | × | × |
| **Experience** | **Professional** | Influence of formal and informal training (i.e., when, why and how should I think through stuff) | × | × | × | × | × | × | × | × |
|  |  | Having time to think (when in the week/month/season/year) | × | × | × |  | × |  | × | × |
|  |  | Balance of careful thinking vs. gut feel decisions in my past | × |  | × |  | × | × |  | × |
|  |  | Decision making (see Meta Process) | × | × | × | × | × | × |  | × |
|  |  | Reflective process (see Reflective Skills) | × | × | × | × | × | × | × | × |
|  | **Reflective Skills** | Internal and external references (coaching team vs. liaison/discussion with others) |  | × |  | × |  | × |  | × |
|  |  | My own skill as a reflective practitioner | × |  |  | × |  |  |  |  |
|  |  | In action (i.e., intuitive basis to reflective practice) | × | × | × | × | × |  | × | × |
|  |  | On-action (i.e., Classic basis for reflective practice) | × |  |  | × | × |  |  | × |
|  |  | On-action/in context (‘auditing’ – quick check on decisions made) | × | × | × | × | × | × | × | × |
|  |  | Pre-action (aspects of planning – how planning of sessions at micro and meso level influence observation, expectation, etc.) | × | × | × |  | × | × | × | × |
| **Meta Process** | **Aspects of Decision Making process** | Considered process, planning, (pre-action, on action, creating time) | × | × | × | × | × | × | × |  |
|  |  | Intuitive process (on-action/in context, creating time) | × | × | × |  |  |  |  | × |
|  |  | In session (in action and on-action/in context, nested) | × | × |  | × |  | × |  |  |
|  |  | Adaptability/flexibility | × | × | × | × | × | × | × | × |
|  |  | Creativity | × | × |  | × | × | × | × | × |
|  | **Audit**  **(change of perspective on DM)** | Meta decision. “How best to make the decision?” | × | × |  | × | × | × | × | × |
|  |  | Personal preference, pros and cons (macro and micro process) | × | × | × | × | × | × | × | × |
